# Past, Present, and Future of Human Rights in Gerontological Education

**DOI:** 10.1093/geroni/igab046.1315

**Published:** 2021-12-17

**Authors:** Judith Howe

**Affiliations:** Icahn School of Medicine at Mount Sinai, Yonkers, New York, United States

## Abstract

The rights of older persons, essential to our work as gerontologists, were discussed in the World Assembly on Aging (1982) and adopted through the United Nations Principles of Older Persons and followed by the Madrid International Plan of Action on Aging (MIPPA) in 2002. Although it has been endorsed by the General Assembly of the United Nations, in contrast to conventions, it is not binding on member states. This paper discusses the rights of older persons and our obligations as educators and researchers to focus on core issues associated with the rights and quality of life of older people. We will review the role of education in meeting this call to action through examples like the UN Decade of Healthy Ageing where education is a required element to accomplish the action areas and the Age-Friendly University movement. Both have involved multiple university communities on a global scale.

